# Benchmark Dose of Urinary Cadmium for Assessing Renal Tubular and Glomerular Function in a Cadmium-Polluted Area of Japan

**DOI:** 10.3390/toxics12120836

**Published:** 2024-11-21

**Authors:** Takuya Hayashi, Kazuhiro Nogawa, Yuuka Watanabe, Teruhiko Kido, Masaru Sakurai, Hideaki Nakagawa, Yasushi Suwazono

**Affiliations:** 1 Department of Occupational and Environmental Medicine, Graduate School of Medicine, Chiba University, Chiba 2608670, Japan; uoeh.hayashi@gmail.com (T.H.); nogawa@chiba-u.jp (K.N.); watanabe155@chiba-u.jp (Y.W.); 2 Division of Health Sciences, Graduate School of Medical Science, Kanazawa University, Kanazawa 9200942, Japan; tkido@staff.kanazawa-u.ac.jp; 3 Department of Social and Environmental Medicine, Kanazawa Medical University, Kanazawa 9200293, Japan; m-sakura@kanazawa-med.ac.jp (M.S.); hnakagaw@kanazawa-med.ac.jp (H.N.)

**Keywords:** benchmark dose, environmental exposure, renal effect, risk assessment, urinary cadmium

## Abstract

The aim of the present study was to apply an updated benchmark dose (BMD) approach to estimate reference urinary cadmium (U-Cd) for renal tubular and glomerular effects. This cross-sectional survey was conducted 30 years ago in 30 men and 44 women living in a Cd-polluted area and in 18 men and 18 women living in a non-polluted area. We applied an updated hybrid approach to estimate the BMDs and 95% lower confidence limits (BMDLs) of U-Cd for creatinine (Cr) clearance (CrCl), estimated glomerular filtration rate (eGFR), β2-microglobulin (β2-MG), and β2-MG tubular reabsorption (%TRβ2-MG). Using a benchmark response (BMR) of 5%, we estimated the BMDLs of U-Cd for adverse renal effect markers to be 2.9 (eGFR), 1.8 (β2-MG), 1.8 (%TRβ2-MG < 95%), and 3.6 μg/g Cr (%TRβ2-MG < 90%) in men, and 3.5 (CrCl), 2.5 (β2-MG), 2.6 (%TRβ2-MG < 95%), and 3.9 μg/g Cr (%TRβ2-MG < 90%) in women. The obtained BMDLs for tubular effects were 1.8–3.6 µg/g Cr and for glomerular effects were 2.9–3.5 µg/g Cr; these are not very high compared to the exposure levels in the general population. The BMDLs calculated in this study provide important information for measures regarding protecting general inhabitants or workers from the adverse health effects of Cd exposure.

## 1. Introduction

Itai-itai disease, the most severe form of chronic cadmium (Cd) poisoning, occurs frequently among the inhabitants of the Jinzu River Basin in Toyama Prefecture. Patients suffer from multiple fractures due to bone damage and renal impairment leading to severe atrophy of the kidneys [1,2]. Itai-itai disease was recognized for the first time in the basin of the Jinzu River around 1920 [2]. Affected patients were typically middle-aged or older women [1,2]. The soil in the region’s rice fields had been contaminated by Cd released from an upstream mine. As a result, rice harvested from these fields had significantly elevated Cd concentrations, resulting in high environmental Cd exposure. In patients with Itai-itai disease, the kidneys were markedly atrophic. Patients with Itai-itai disease had an average kidney weight of 40 g [3], whereas the average normal kidney weight is 120 g to 150 g. Histopathological examination revealed atrophic, desquamated, or absent proximal tubules and the majority of glomeruli being extinct, with so-called hyalinized glomeruli or global glomerular sclerosis [3]. The results of many previous studies have shown that long-term exposure to Cd causes renal dysfunction [1,4]. It is also well known that tubular damage occurs first, followed by glomerular damage and further bone effects [1,4]. After Friberg’s report [5] described how the proteinuria found in Cd workers consisted mainly of low-molecular-weight proteins, a number of studies have indicated that Cd-induced renal damage is characterized by proximal tubular renal reabsorption dysfunction [6]. The first signs of Cd-induced renal damage are elevated urinary excretion of low-molecular-weight proteins, particularly β2-microglobulin (β2-MG) and α1-microglobulin. The urinary excretion of β2-MG in subjects with Cd-induced renal damage is in proportion with the severity of damage [6]. Levels of 100,000 μg/g of creatinine (Cr) can be reached in cases of severe tubular damage, such as in cases of Itai-itai disease. In addition, it may progress to glomerular damage with decreased glomerular filtration rate, as reported in studies of occupationally exposed workers [4,7]. Several studies in environmentally exposed populations suggest that reduced glomerular filtration rate and creatinine clearance (CrCl) and elevated serum creatinine may occur at cadmium doses similar to those of tubular injury [4,8].Thus, it is crucial to establish reference exposure levels that do not cause early renal damage in order to prevent health complications associated with Cd, which, if severe, could lead to Itai-itai disease.

As a way of setting such reference values, the benchmark dose (BMD) method has been used to assess the health risks of environmental contaminants [9,10]. The BMD is defined as the exposure level that corresponds to a specific increase in the probability of an adverse response (benchmark response, BMR) compared to background exposure. The lower bound of the 95% confidence limit of the BMD (BMDL) can be used in risk assessment as a replacement for the no observed adverse effect level [9,10].

Therefore, in the Japanese population, BMDLs of urinary cadmium (U-Cd), adopted as an exposure indicator for Cd body burden, have been calculated in relation to renal damage by applying the BMD method [11]. These studies have targeted the effects of U-Cd on renal tubular indices, and no results are available for Japanese individuals with regard to renal glomerular damage. To our knowledge, there are very few reports in other populations, with published findings only for those in Sweden [12] and Thailand [13].

Estimations of BMD and BMDL for continuous outcomes have been developed using the hybrid approach [12,14,15]. For continuous dose–response data, a number of proposals have been made for the definition of BMD. The hybrid approach defines BMD as corresponding to some increase in the probability of falling below (or exceeding) a cut-off representing an ‘adverse’ level of continuous response [14,16,17,18]. The hybrid concept suggests a generalization of the BMD concept by allowing the use of same response definitions originally proposed for quantitative data [18]. With this method, BMD and BMDL were estimated based on continuous exposure and a continuous effect marker, thereby avoiding categorization of subjects [12,14,15]. Accordingly, the statistical validity and efficiency of BMD and BMDL were higher with the hybrid approach compared with methods involving categorization of continuous exposure and effect markers.

Environmental Cd pollution also resulted from mining activities in the Kakehashi River Basin, Ishikawa Prefecture, while a large-scale epidemiological study conducted in 1974 and 1975 revealed a high incidence of renal tubular disorders among inhabitants. Since then, regular health check-ups have been carried out, and more detailed check-ups in terms of renal function, such as CrCl, have continued to be carried out, particularly for those with concerns about renal dysfunction [19]. Cr is an endogenous substance that is mainly excreted by the glomeruli of the kidney. CrCl has traditionally been used as a renal function test and as an indicator of renal glomerular function. A portion of results from those assessments have already been reported [20].

The aim of the present study was to apply an updated BMD approach to estimate reference U-Cd values for Cd-induced renal effects, including tubular and glomerular effects, based on a dataset collected during the previous investigation [20].

## 2. Materials and Methods

### 2.1. Study Population and Measurement

This cross-sectional survey was conducted 30 years ago, and some of the results have already been reported [20]. Based on these data, we applied the recently developed BMD method. The subjects in this study consisted of 30 men and 44 women, all of whom were over 50 years of age and lived in the Cd-polluted Kakehashi River Basin in Ishikawa Prefecture in 1985. They all showed Cd-induced renal tubular dysfunction and were officially recognized as ‘subjects requiring observation’ by the research committee organized by the Prefectural Health Authority [19]. In this area, Cd compounds were carried by the Kakehashi River from a mine upstream to rice fields, where river water was used for irrigation. As non-Cd-exposed subjects, 18 men and 18 women over 50 years of age living in a non-Cd-polluted area were selected and underwent health checkups. We obtained 2-hour urine samples from each participant. Blood specimens were drawn at the midpoint of the collection period. β2-MG levels in serum and urine were analyzed by the Phadebas β2-microtest (Pharmacia, Upsala, Sweden). Cr concentrations in serum and urine were determined by Jaffe’s method [21]. U-Cd was determined directly by performing graphite-furnace atomic absorption spectrometry with a Hitachi Model Z-8100 (Hitachi, Tokyo, Japan). The survey was conducted in the 1980s, and verbal consent to participate was obtained at that time [20]. For re-analyzing the previous data, the study protocol was re-approved by the Ethics Review Board of the Graduate School of Medicine, Chiba University.

### 2.2. Statistical Analysis

The concentrations of urinary analytes were expressed in corrected Cr units (/g Cr). CrCl was calculated with the following formula:

$$CrCl\;(mL/min)=\frac{U\text{-}Cr\;(mg/mL)\times UV\;(mL)}{S\text{-}Cr\left(mg/dL\right)\times 0.01\;(1\;dL/100\;mL)\times 2\;(h)\times 60\;(min)}\times \frac{1.73\;(m^{2})}{BSA\;(m^{2})}$$

where U-Cr = creatinine concentration in a 2-h urine sample; UV = a 2-h urinary volume; S-Cr = serum creatinine concentration; and BSA = body surface area calculated with the formula by Du Bois [22].

The estimated glomerular filtration rate (eGFR, mL/min/1.73 m^2^) was calculated with the following formula, which was established by the Japanese Society of Nephrology for Japanese individuals [23,24]:

$$eGFR=194\;\times \;S\text{-}{Cr\;(mg/dL)}^{-1.094}\;\times \;{Age}^{-0.287}(\times \;0.739\;if\;women)$$

This formula employs the S-Cr values measured by the enzymatic method. Since the S-Cr measured by the Jaffe method has been reported to be 0.2 mg/dL higher than that measured by the enzymatic method [25], we substituted S-Cr-0.2 mg/dL for the ‘S-Cr’ term in this formula.

The fraction of tubular reabsorption of filtered phosphorus (%TRP) has often been used as an indicator of tubular function. In this study, as the indicator of tubular reabsorption, the concentration of β2-MG was applied instead of phosphorus to calculate the fraction of tubular reabsorption of filtered β2-MG (%TRβ2-MG) with the following formula:

$$\%TR\beta 2\text{-}MG=\left(1-\frac{U\text{-}\beta 2\text{-}MG\;(µg/L)\times S\text{-}Cr\;(mg/dL)\times 0.01\;(dL/100mL)}{S\text{-}\beta 2\text{-}MG\;(µg/L)\times U\text{-}\mathrm{Cr}\;(mg/mL)}\right)\times 100$$

β2-MG freely passes through the glomerulus, and more than 99% is reabsorbed by the proximal renal tubules [26]. Thus, we adopted %TRβ2-MG as the indicator of tubular reabsorption, as well as %TRP. We did not treat this reabsorption rate as a continuous variable, due to its limited range between 0% and 100%. Cut-off values of 95% and 90% were adopted to dichotomize the %TRβ2-MG level as the outcome. CrCl, eGFR, and U-β2-MG (log transformed to obtain a normal distribution) were treated as continuous variables of the renal glomerular and tubular effects.

We determined the BMD/BMDL of U-Cd for continuous variables using the hybrid approach [12], and for dichotomous %TRβ2-MG using logistic regression analysis, adjusting for the age effect by including it into the statistical model. The BMR was set at 5% or 10% additional risk above the background exposure. If the U-Cd was not significant, no further estimation of the benchmark dose was performed due to the reliability of the results.

The analyses were performed with IBM SPSS19J (IBM Business Analytics, Tokyo, Japan) and Microsoft Excel 2021 (Microsoft Corporation, Redmond, WA, USA). A *p*-value < 0.05 was considered statistically significant.

## 3. Results

The main characteristics of the participants are presented in Table 1. Their average age was around 70 years for both sexes. Most subjects were those in need of observation in Cd-polluted areas, and their U-Cd, β2-MG, and prevalence of low %TRβ2-MG (<95%, <90%) tended to be higher than those from non-polluted area. On the other hand, CrCl, eGFR, and %TRβ2-MG tended to be lower in those from Cd-polluted area than in those from non-polluted area. Regarding the prevalence of %TRβ2-MG, 35% of participants were below 95% and around 20% were below 90%, suggesting a tubular effect of Cd exposure on the reabsorption of low-molecular-weight proteins.

**Figure 1:**
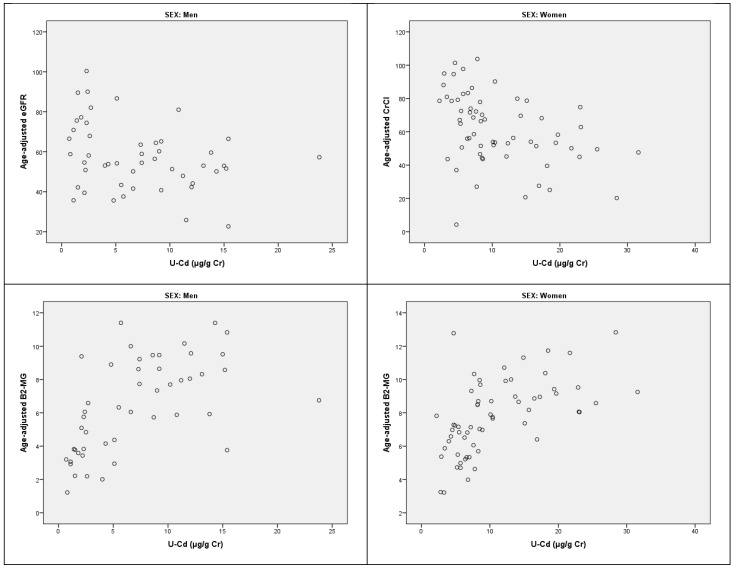
Associations between U-Cd and age-adjusted (70 years) markers of renal function, according to the results of multiple regression analyses (significant items only).

Table 2 shows the results of multiple linear regression analyses and logistic regression between U-Cd and indicators of renal function, grouped according to gender. U-Cd was significantly related to CrCl in women, to eGFR in men, and to log-transformed β2-MG and low %TRβ2-MG (<95%, <90%) in both men and women. Figure 1 shows the associations between U-Cd and age-adjusted (70 years) markers of renal function, according to the results of multiple regression analyses (significant items only).

Table 3 shows BMDs and BMDLs of U-Cd for renal markers. By using a BMR of 5%, we estimated BMDLs of U-Cd for renal effect markers to be 2.9 (eGFR), 1.8 (β2-MG), 1.8 (%TRβ2-MG < 95%), and 3.6 μg/g Cr (%TRβ2-MG < 90%) in men, and 3.5 (CrCl), 2.5 (β2-MG), 2.6 (%TRβ2-MG < 95%), and 3.9 μg/g Cr (%TRβ2-MG < 90%) in women.

## 4. Discussion

In this study, for the first time, we found the relationship between U-Cd and renal glomerular effects in a population of Japanese residents, and we were also able to establish the relationship between U-Cd, U-β_2_-MG, and tubular reabsorption. Furthermore, the new BMD method was applied to the previous results, and new knowledge was obtained for future discussions on the health effects of Cd exposure, based on valuable data that are not presently available. Furthermore, BMDLs for U-Cd were estimated as reference values for renal glomerular and tubular effects in residents of Cd-polluted and non-polluted areas in Japan. The BMDLs obtained ranged from 1.8 to 3.6 µg/g Cr for tubular effects and 2.9–3.5 µg/g Cr for renal glomerular effects. As mentioned above, no reference values of U-Cd have been obtained for renal glomerular effects in the Japanese population. Therefore, we believe that such values are obtained for the first time by re-analyzing present data.

BMDLs/BMDs of U-Cd for tubular and glomerular effects were estimated in 820 Swedish women aged 53–64 years [12]. The BMDL (BMD) for eGFR inferred from serum cystatin C concentrations was calculated as 0.7 (1.1) μg/g Cr, with a BMR of 5%, adjusted for age and other potential covariates. This value was considerably lower than expected and very close to the BMDL (BMD) for tubular effects [0.5 (0.6) μg/g Cr] in that study. As for the glomerular effects in this study, the BMDL (BMD) was relatively high at 2.9–3.5 (5.7) μg/g Cr, compared to those in Swedish women. However, it was very close to the BMDL (BMD) for the tubular effect (β_2_-MG) of 1.8–3.9 (2.6–5.4) μg/g Cr, confirming that glomerular damage due to Cd exposure occurs earlier than expected at levels similar to those that induce a renal tubular effect in Japanese and Swedish individuals.

Furthermore, the BMDL was also estimated in 289 men and 445 women from Bangkok (a low-exposure area), as well as in individuals from Cd-contaminated areas of the Mae Sot District (a high-exposure area) in Thailand [13]. In target participants, 42.8% were smokers, while 31.7% had hypertension, and 9% had chronic kidney disease (CKD, eGFR ≤ 60 mL/min/1.73 m^2^). In that study, the BMDL for CKD as a glomerular outcome was 3.26 µg/g Cr in men and 4.98 µg/g Cr in women. In the present study, with a BMR of 10%, the BMDL was 5.9 µg/g Cr for men and 4.9 µg/g Cr for women, which is in close agreement with that study. We consider that the reference exposure to protect against glomerular effects in Asian individuals can be further established in the present study. In that study, the BMDLs for tubular damage (β2-MG ≥ 300 µg/g Cr) were 0.469 µg/g Cr for men and 0.733 µg/g Cr for women. BMDLs for β2-MG, as continuous variables, were also calculated in the present study using the hybrid approach. Thus, the difference in estimation methods may be the reason for this difference between the present estimates and the estimates in that study. The BMDLs of U-Cd for tubular effects in the present study were 1.8 (men) and 2.5 µg/g Cr (women) for β2-MG, and 1.8 (men) and 2.6 µg/g Cr (women) for %TRβ2-MG. These results indicate that both tubular and glomerular effects may occur at usual exposures in the general Asian population.

Another notable feature of the present study was that the tubular reabsorption of β2-MG could be evaluated as tubular damage. This low-molecular-weight protein readily passes through the glomerular membrane; subsequently, more than 99.9% of the filtered β2-MG is reabsorbed and degraded in the proximal tubules, with only about 5 µg/h of the protein appearing in the final urine [26]. In the present study, in addition to the urinary β2-MG concentration, the serum β2-MG concentration was also measured, allowing the reabsorption rate of β2-MG to be calculated, and a quantitative assessment of tubular function to be performed. Although no report on the reabsorption rate of β2-MG has been found so far, and only an approximate cut-off could be adopted in the present study, we consider that the dose–response relationship between U-Cd and tubular function was determined for the first time, and the present results add a new and useful insight into the tubular effects of Cd exposure.

Previously, the Scientific Panel on Contaminants in the Food Chain (CONTAM) of the European Food Safety Authority (EFSA) carried out a meta-analysis of data from 35 studies based on 30,000 individuals using the BMD method with the hybrid approach [27]. When the BMR was set at 5%, the estimated BMDL was 4 μg/g Cr for β_2_-MG in that evaluation [27]. Furthermore, the Joint FAO/WHO Expert Committee on Food Additives (JECFA) has also continued to evaluate Cd exposure worldwide. At the 33rd JECFA meeting, the provisional tolerable weekly intake (PTWI) of 7 μg Cd/kg of body weight was established, in consideration of safety margins, based on the evidence that renal tubular damage begins to occur when the renal cortical Cd concentration exceeds 200 mg/kg [28]. Furthermore, at the 55th JECFA meeting, the PTWI of 7 μg Cd/kg of body weight was maintained, with the assessment that tubular damage begins to occur at U-Cd levels above 2.5 μg/g Cr, based on information from a meta-analysis at that time [29]. Subsequently, at the 63rd JECFA, using data from a meta-analysis conducted at EFSA [27], the investigators concluded that β2-MG increased rapidly above a U-Cd of 5.24 μg/g Cr, based on data from over 30,000 participants [30]. That result is also considered by JECFA in the review of tolerable intake of Cd in the general population. The BMDLs in the present study are also very close to the reference values evaluated by the EFSA and JECFA. We believe that we have clarified the usefulness and reliability of tolerable intake of Cd by the EFSA or JECFA for protection against glomerular and tubular effects of environmental Cd exposure in the general population.

One limitation of this study is that the sample size was small, not reaching 100 for both sexes. Conversely, since the subjects were selected from Cd-polluted and non-polluted areas, the range of U-Cd indicating environmental Cd exposure was wide, and the dose–effect and dose–response relationships could be evaluated broadly, which addresses the sample size problem to some extent. On the other hand, biomarkers of kidney effects are influenced by gender, age, body mass index, physical exercise, and diurnal variation, and these factors can either confound or modify dose–effect and dose–response relationships. However, due to the lack of information on these covariates, other than sex and age, corrections for them could not be made in the present study. It will therefore be necessary to take this into account when interpreting the results. In addition, the measured results are from the past, and the exposure levels are likely to be very different from those of the general population today. However, the BMDLs obtained as reference levels are also very close to the U-Cd levels observed in general inhabitants and workers around the world today, and they can still be considered useful in the assessment of the health effects of current Cd exposures.

## 5. Conclusions

In the present study, health survey data collected during the 1980s from inhabitants of Cd-polluted and non-polluted areas were re-evaluated when the Cd exposure was still considerably large. The obtained BMDLs for tubular effects were 1.8–3.6 µg/g Cr and glomerular effects were 2.9–3.5 µg/g Cr, which are not very high compared with the exposure levels in the general population. The BMDLs calculated in this study provide important additional information for the future assessment of Cd exposure in general inhabitants and workers, as well as provide considerations for preventive measures against the health effects caused by Cd exposure.

## Figures and Tables

**Table 1:** Characteristics of the participants and exposure data and markers of renal function, grouped according to area and gender.

	Cd-Polluted Area		Non-Polluted Area		All	
	Men (N = 30)	Women (N = 44)	Men (N = 18)	Women (N = 18)	Men (N = 48)	Women (N = 62)
Variable	Mean (SD ^1^)	Mean (SD ^1^)	Mean (SD ^1^)	Mean (SD ^1^)	Mean (SD ^1^)	Mean (SD ^1^)
Age, y	74.1 (7.8)	73.2 (7.2)	62.9 (9.9)	63.9 (9.0)	69.9 (10.1)	70.5 (8.8)
CrCl, mL/min	52.8 (23.0)	48.6 (17.8)	96.7 (30.3)	90.9 (18.4)	69.3 (33.5)	60.9 (26.3)
eGFR, mL/min	46.9 (12.4)	46.7 (17.7)	75.5 (18.4)	83.7 (15.8)	58.1 (20.6)	58.1 (24.5)
	**GM ^2^ (GSD ^3^)**	**GM ^2^ (GSD ^3^)**	**GM ^2^ (GSD ^3^)**	**GM ^2^ (GSD ^3^)**	**GM ^2^ (GSD ^3^)**	**GM ^2^ (GSD ^3^)**
U-Cd, μg/g Cr	9.0 (1.6)	11.6 (1.7)	1.9 (1.7)	4.8 (1.5)	5.0 (2.5)	9.0 (1.9)
U-β2-MG, μg/g Cr	5537.9 (7.2)	10,029.4 (5.9)	14.8 (6.5)	95.6 (7.1)	600.3 (32.2)	2597.5 (16.4)
%TRβ2-MG ^4^, %	91.0 (1.1)	82.7 (1.4)	100.0 (1.0)	99.8 (1.0)	94.3 (1.1)	87.4 (1.4)
**Prevalence of Decreased %TRβ2-MG ^4^**	**N (%)**	**N (%)**	**N (%)**	**N (%)**	**N (%)**	**N (%)**
%TRβ2-MG ^4^ < 95%	16 (53.3%)	23 (52.3%)	0 (0.0%)	0 (0.0%)	16 (33.3)	23 (37.1)
%TRβ2-MG ^4^ < 90%	9 (30.0%)	16 (36.4%)	0 (0.0%)	0 (0.0%)	9 (18.8)	16 (25.8)

^1^ Standard deviation. ^2^ Geometric mean. ^3^ Geometric standard deviation. ^4^ % Tubular reabsorption of β2-microglobulin.

**Table 2:** Results of multiple linear regressions and logistic regression analyses between U-Cd and markers of renal function, grouped according to gender.

Renal Effect Marker		Men		Women	
Multiple Regression Analysis	Explanatory Variable	B ^1^ (95% CI ^2^)	*p*	B ^1^ (95% CI ^2^)	*p*
CrCl (mL/min)	U-Cd, μg/g Cr	−1.37 (−3.11, 0.36)	0.118	−1.24 (−1.99, −0.48)	0.002
	Age, y	−1.32 (−2.23, −0.41)	0.005	−1.55 (−2.14, −0.96)	<0.001
eGFR (mL/min)	U-Cd, μg/g Cr	−1.02 (−2.01, −0.04)	0.041	−0.65 (−1.39, 0.09)	0.082
	Age, y	−0.97 (−1.49, −0.46)	<0.001	−1.56 (−2.14, −0.98)	<0.001
β2-MG (μg/g cr) ^3^	U-Cd, μg/g Cr	0.30 (0.16, 0.44)	<0.001	0.18 (0.11, 0.25)	<0.001
	Age, y	0.15 (0.08, 0.23)	<0.001	0.16 (0.11, 0.22)	<0.001
**Logistic Regression Analysis**	**Explanatory Variable**	**OR ^4^ (95% CI ^2^)**	***p***	**OR ^4^ (95% CI ^2^)**	***p***
%TRβ2-MG ^5^ (<95%)	U-Cd, μg/g Cr	1.17 (1.02, 1.35)	0.022	1.17 (1.05, 1.29)	0.003
	Age, y	1.04 (0.97, 1.13)	0.280	1.08 (1.00, 1.17)	0.061
%TRβ2-MG ^5^ (<90%)	U-Cd, μg/g Cr	1.21 (1.03, 1.42)	0.021	1.12 (1.02, 1.23)	0.017
	Age, y	1.07 (0.96, 1.18)	0.213	1.09 (1.00, 1.20)	0.057

^1^ Regression coefficients. ^2^ 95% confidence interval. ^3^ β2-MG naturally log-transformed. ^4^ Odds ratio. ^5^ % Tubular reabsorption of β2-microglobulin.

**Table 3:** Benchmark doses (BMDs) of U-Cd with their lower limits (BMDLs) for markers of renal functions, calculated using the hybrid approach and logistic regression analysis.

			BMR = 5%	BMR = 10%
Hybrid Approach	P (0) ^1^	Cut-Off Value ^2^	BMDL (BMD)	BMDL (BMD)
CrCl (women)	5.0%	43.3 mL/min	3.5 (5.7) μg/g Cr	5.9 (9.6) μg/g Cr
eGFR (men)	5.0%	38.8 mL/min	2.9 (5.7) μg/g Cr	4.9 (9.6) μg/g Cr
U-β2-MG (men)	5.0%	3094 μg/g Cr	1.8 (2.8) μg/g Cr	3.1 (4.6) μg/g Cr
U-β2-MG (women)	5.0%	6194 μg/g Cr	2.5 (3.6) μg/g Cr	4.2 (6.0) μg/g Cr
**Logistic Regression Analysis**	**Cut-Off Value**	**P (0) ^3^**	**BMDL (BMD)**	**BMDL (BMD)**
%TRβ2-MG (men)	<95%	11.8%	1.8 (2.6) μg/g Cr	3.1 (4.6) μg/g Cr
%TRβ2-MG (women)	<95%	8.1%	2.6 (3.5) μg/g Cr	4.5 (6.0) μg/g Cr
%TRβ_2_-MG (men)	<90%	3.4%	3.6 (5.0) μg/g Cr	5.6 (7.7) μg/g Cr
%TRβ_2_-MG (women)	<90%	6.6%	3.9 (5.4) μg/g Cr	6.6 (9.1) μg/g Cr

^1^ Definition of background prevalence rates. ^2^ The estimated cut-off values for each renal effect marker based on the defined background prevalence rates and the results of multiple regression analyses. ^3^ The estimated background prevalence rate based on the results of the logistic regression analyses.

## Data Availability

The data presented in this study are available upon request from the corresponding author due to privacy restrictions.

## Abbreviations

BMD	Benchmark dose
BMDL	Benchmark dose low
BMR	Benchmark response
Cd	Cadmium
CKD	Chronic kidney disease
Cr	Creatinine
CrCl	Creatinine clearance
eGFR	Estimated glomerular filtration rate
EFSA	European Food Safety Authority
%TRP	Fraction of tubular reabsorption of filtered phosphorus
%TRβ2-MG	Fraction of tubular reabsorption of filtered β2-MG
JECFA	Joint FAO/WHO Expert Committee on Food Additives
PTWI	Provisional tolerable weekly intake
CONTAM	Scientific Panel on Contaminants in the Food Chain
U-Cd	Urinary cadmium
β2-MG	Β2-microglobulin
